# Physiologically motivated multiplex Kuramoto model describes phase diagram of cortical activity

**DOI:** 10.1038/srep10015

**Published:** 2015-05-21

**Authors:** Maximilian Sadilek, Stefan Thurner

**Affiliations:** 1 Section for Science of Complex Systems, Medical University of Vienna, Spitalgasse 23, A-1090 Vienna, Austria; 2 Santa Fe Institute, 1399 Hyde Park Road, New Mexico 87501, USA; 3 IIASA, Schlossplatz 1, A-2361 Laxenburg, Austria

## Abstract

We derive a two-layer multiplex Kuramoto model from Wilson-Cowan type physiological equations that describe neural activity on a network of interconnected cortical regions. This is mathematically possible due to the existence of a unique, stable limit cycle, weak coupling, and inhibitory synaptic time delays. We study the phase diagram of this model numerically as a function of the inter-regional connection strength that is related to cerebral blood flow, and a phase shift parameter that is associated with synaptic GABA concentrations. We find three macroscopic phases of cortical activity: background activity (unsynchronized oscillations), epileptiform activity (highly synchronized oscillations) and resting-state activity (synchronized clusters/chaotic behaviour). Previous network models could hitherto not explain the existence of all three phases. We further observe a shift of the average oscillation frequency towards lower values together with the appearance of coherent slow oscillations at the transition from resting-state to epileptiform activity. This observation is fully in line with experimental data and could explain the influence of GABAergic drugs both on gamma oscillations and epileptic states. Compared to previous models for gamma oscillations and resting-state activity, the multiplex Kuramoto model not only provides a unifying framework, but also has a direct connection to measurable physiological parameters.

Fast electrochemical processes taking place on a complicated cytoarchitectural network structure render the human brain a highly complex dynamical system. Brain activity, as measured directly via EEG or MEG, or indirectly by means of MRI recordings, reveals characteristic macroscopic patterns such as oscillations in various frequency bands^1^, synchronization^2^,^3^,^4^, or chaotic dynamics^5^. Generally it is believed that macroscopic activity (involving 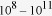 neurons) is closely related to high-level functions such as cognition, attention, memory or task execution. To understand the mechanisms of this correspondence, both the overall network structure of the brain and the local properties of neural populations have to be taken into account^4^,^6^. Regarding the latter, neural inhibition seems to be essential for cortical processing^7^.

Two physiological phenomena received much attention lately in terms of a mathematical understanding: resting-state activity, and gamma oscillations. Resting-state activity is spontaneous, highly structured activity of the brain during rest, and can be described in terms of networks of simultaneously active brain regions^8^,^9^. Models of resting-state networks often rely on anatomical networks derived from histological or imaging data, and on local interactions between populations of excitatory and inhibitory neurons^10^,^11^,^12^,^13^. Oscillatory neural activity in the gamma range (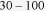 Hz) is potentially related to consciousness and the binding problem although its precise function remains unclear^14^. To understand the origin of gamma oscillations, two mechanisms have been proposed^15^. One describes interactions between inhibitory neurons together with an external driving force^16^,^17^. The other mechanism is based on excitatory-inhibitory coupling with synaptic time delays^18^,^19^,^20^. The relation of gamma oscillations and inhibition is experimentally well established. In mice^21^, rats^22^ and humans^3^,^23^, a decrease of GABA-concentrations (gamma-aminobutyric acid is the main inhibitory neurotransmitter in mammals) is accompanied by a strong attenuation of the gamma frequency band and sometimes by epileptiform activity.

Many existing network models for resting-state activity and gamma oscillations are based on single-neuron local dynamics^10^,^11^,^16^,^17^,^18^,^19^. Since experimentally observed resting-state networks comprise individual regions containing about 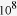 to 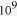 individual neurons, we believe that a local description in terms of Wilson-Cowan equations is an attractive alternative. The subject of multiplex networks received recent attention with applications reaching from social and technological systems to economy and evolutionary games^24^,^25^.

In this work we derive a simple two-layer multiplex model from classical physiological equations that is able to capture the main features of cortical activity such as oscillations, synchronization and chaotic dynamics. This model unifies the roles of neural network topology, synaptic time delays, and excitation/inhibition. It provides a closed framework for simultaneously understanding the origin of resting-state activity and gamma oscillations.

## Results

### Derivation of the multiplex Kuramoto model

**Figure 1:**
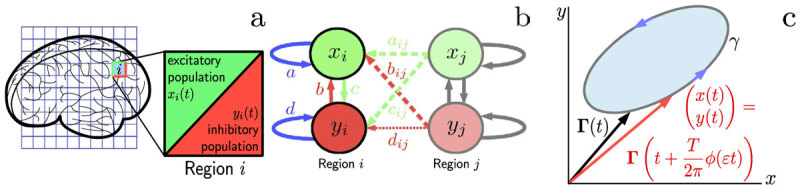
Schematic illustration of the model setup. **a** The cortical surface is divided into 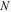 macroscopic regions. Every region 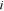 (blue) comprises excitatory (green) and inhibitory (red) neural populations with activity levels 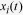 and 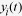, respectively. Activity levels quantify the ratio of firing neurons in the region at time 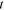. **b** Region 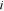 (left) receives excitatory (green arrows) and inhibitory (red arrows) inputs plus self-feedback (blue arrows). Inputs from adjacent regions 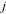 (right) are weak (dashed arrows) or very weak (dotted arrow). **c** Variable transformation from activity variables 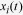 and 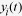 to phase deviation variables 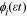. On a limit cycle 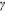, 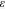-perturbations of the 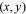-dynamics at time 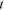 induce the phase deviations 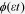.

We consider 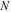 cortical regions indexed by 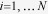, see Fig. 1a. Each region is populated by ensembles of excitatory and inhibitory neurons (e.g. pyramidal cells and interneurons). We define the activity level of a region 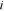 as the fraction of firing excitatory (inhibitory) neurons of the total number of excitatory (inhibitory) neurons in that region at a unit time interval, and denote it by 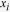 (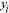). Neglecting for the moment interactions between different regions, we assume that individual cortical regions obey the Wilson-Cowan-type dynamics^20^
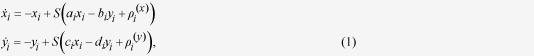
where 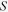 is a sigmoidal response function, 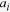 and 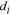 are real-valued feedback parameters, and 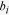 and 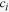 are positive synaptic coefficients (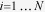). 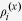 and 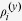 account for external inputs, e.g. from sensory organs. We now introduce interactions among regions of the network by replacing 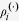 in Eq. (1) by
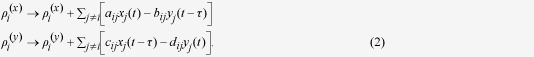
Here 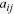, 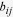, 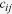, 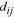 are positive synaptic coefficients linking regions 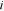 and 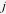. 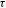 accounts for transmission delays at inhibitory synapses (not to be confused with axonal conduction delays). In physiology, 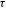 can be altered by changing the synaptic concentration of GABA^18^,^19^,^21^,^22^. In the present model, we assume that 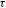 is proportional to the average synaptic GABA concentration in the brain. To derive a multiplex Kuramoto model (MKM) from Eqs. (1) and (2), we make the following three assumptions:

(*i*) *Homogeneity.* Cortical regions exhibit nearly identical dynamical behavior. We therefore assume the following parameters to be constant across regions,
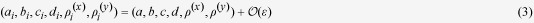
for all 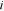, up to small perturbations, denoted by 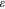.

(*ii*) *Stable local oscillations.* We choose the parameters 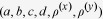 such that each uncoupled system Eq. (1), under the assumption given in Eq. (3), has a unique exponentially stable limit cycle 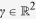. As a consequence, after a transient time solutions of Eq. (1) can be written as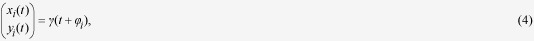 where 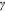 (*t*) is an arbitrary solution of Eq. (1) on 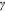
26,27. 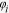 accounts for specific initial values. Let 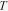 denote the period of 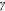. We assume that the frequency 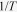 lies in the physiological gamma range.

(*iii*) *Weak coupling.* Interactions between adjacent regions are weak, and inhibitory-inhibitory interactions are very weak in the sense that,
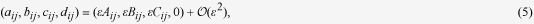
for all 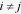. These assumptions are justified because the number of synaptic connections within a cortical region is much larger than between regions, and excitatory neurons outnumber inhibitory neurons by approximately one order of magnitude^28^,^29^.

Figure 1b summarizes the connectivity structure between regions 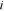 and 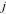. Region 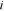 receives excitatory (green arrows) and inhibitory (red arrows) inputs plus feedback (blue arrows), magnitudes are indicated by the arrow labels. For the sake of clarity, arrows representing inputs of magnitude 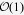, 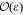 and 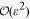 are drawn in continuous, dashed and dotted style, respectively.

Under these assumptions, the system Eq. (1) with Eq. (2) is equivalent (see SI) to a two-layer MKM
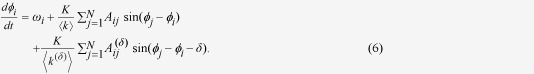
Here 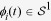 describes the deviations from the uncoupled phases 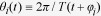 that are associated with solutions of the uncoupled system Eq. (4). Accordingly, 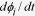 describes the deviations from the uncoupled oscillation frequency 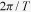. Time 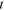 has been rescaled, see SI. 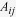 is the adjacency matrix of the excitatory-excitatory interaction network as defined in Eq. (5), and 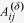 is a linear combination of the adjacency matrices 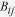 and 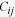. 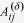 accounts for the interaction between excitatory and inhibitory populations, see SI. 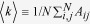, and 
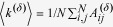
, are the corresponding average degrees. 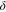 is a phase shift parameter related to the time delay 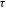 via 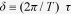. 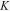 is a global coupling constant that we assume to be proportional to the cerebral blood flow. This is reasonable because the latter is strongly correlated with the connection strengths of functional networks reconstructed in magnetic resonance imaging^30^. 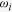, the so-called *natural frequencies* of the MKM, are the constant contribution to the frequency deviations 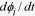. We take 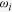 from a symmetric, unimodal random distribution 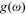, with mean 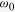. Since the 1-parameter family of rotating-frame transformations 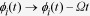, 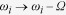, leave Eq. (6) invariant for any 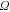, without loss of generality we assume, 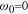 and 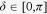. Note that for each solution 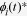 with 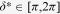, there exists a solution 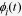 with 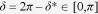 and 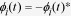. Physiological processes changing 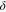, 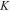, and 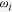 occur on a much slower timescale than neural activity.

It is known that weakly coupled, nearly identical limit-cycle oscillators can be described in terms of phase variables^27^,^31^,^32^,^33^. However, in terms of the new variables 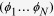, interactions between cortical regions take place on two independent layers representing excitatory-excitatory and excitatory-inhibitory coupling, respectively, and the complicated connectivity structure of Fig. 1b reduces to a simple two-layer multiplex structure. Figure 1c shows the variable transformation from activity variables 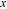 and 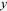, to the phase variable 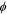 for any cortical region. In the unperturbed case, 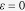, the limit cycle 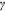 is parametrized by 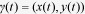. Since 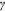 is exponentially stable, 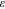-perturbations of activity dynamics 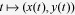 lead to phase deviations 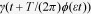. For 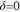 we recover the Kuramoto model on a single network, see SI and^34^,^35^,^36^,^37^,^38^,^39^.

### Order parameters

We characterize solutions of Eq. (6) by the following order parameters:

#### Synchronization

We define the order parameter^32^,^33^,^34^,^40^
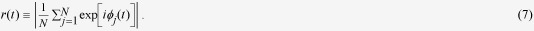


It takes values between 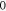 (no synchronization) and 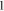 (full synchronization)^27^. Let 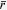 denote its time average 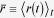.

#### Chaotic dynamics

The instantaneous largest Lyapunov exponent is given by
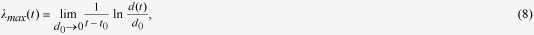
where 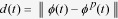 measures the separation between a reference trajectory 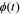 and a perturbed one 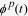. 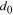 is the initial separation at 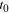, and 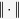 is the 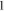-norm, see SI. For large times, 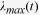 approaches the “true” largest Lyapunov exponent, 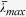.

#### Average frequency deviation

We look at average frequency deviations across all regions,
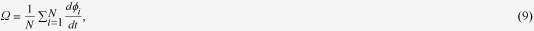
once a stationary state is reached.

### Numerical simulation of the model

#### Synchronization

**Figure 2:**
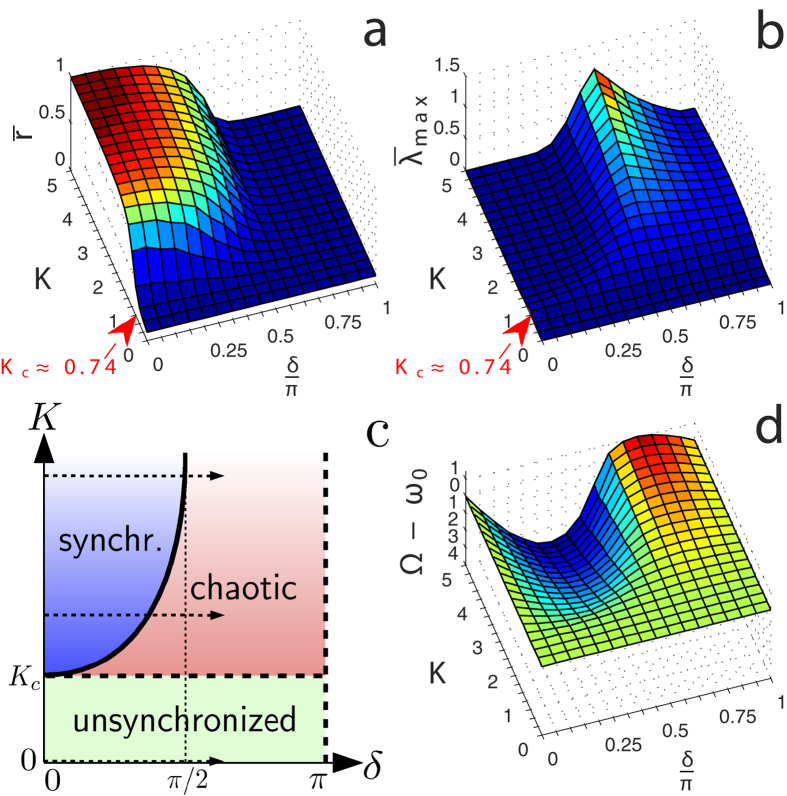
Dynamical properties of the multiplex Kuramoto model in terms of control- and order parameters as obtained by numerical simulation. **a** Order parameter 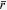 and **b** largest Lyapunov exponent 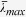 identify the mutually exclusive regions of synchronization and of chaotic dynamics in the 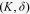-plane. The critical point (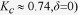 indicates a phase transition of Kuramoto type in the case of vanishing synaptic time delays. The region 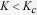 is characterized by 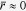 and 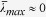. For 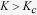, either 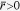 and 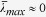 (synchronized region) or 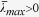 and 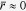 (chaotic region). Within the chaotic region, the smallest values of 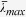 are encountered at 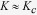, where 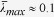. The largest values of 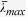 occur at the boundary with the synchronized region, with peak values of 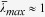. **c** Schematic phase diagram inferred from **a** and **b**. Synchronized, unsynchronized and chaotic behavior can be clearly distinguished. Dashed arrows indicate directions along which distributions of frequency deviations were evaluated in Fig. 3d. Average frequency deviations 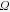 in the 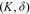-plane. Regions of frequency suppression, 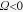, have a large overlap with the synchronized phase.

We find that synchronization 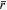 depends on the coupling strength 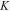, and phase shift 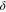, Fig. 2a. For 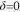, we expect (see SI) a transition from an unsynchronized to a synchronized state at a critical value 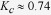, which is confirmed by our simulations, Fig. 2a. With 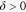, stronger coupling 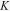 is required for this transition to occur. Above a value of approximately 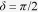, a global synchronized state ceases to exist.

#### Chaotic dynamics

Above the synchronization threshold, 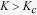, synchronization and chaotic dynamics are mutually exclusive, see Fig. 2b. For small values of 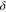, there exists a small chaotic region (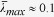) at the Kuramoto transition, in agreement with the well-known results for 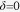, see SI. This region is expanding with increasing values of 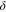. At the boundary to the synchronized region, increasingly large values of 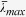 are obtained. 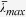 peaks at 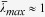, for 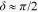. In the unsynchronized region, 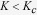, the dynamics is not chaotic, 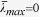. For 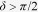 and 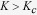, which constitutes the largest fraction of the chaotic region, the smallest values of Lyapunov exponents that we obtain are between 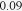 and 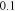. Those values typically occur close to the border to the unsynchronized region, where 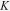 is close to 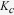. For comparison, we note that at the classical Kuramoto transition (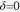 and 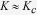), where chaotic behavior of the system is out of question^35^, values of maximally 0.07 are encountered in our model set-up. Figure 2c integrates both results (synchronization and chaotic dynamics) into a schematic phase diagram that clearly exhibits three phases.

#### Spectral properties

**Figure 3:**
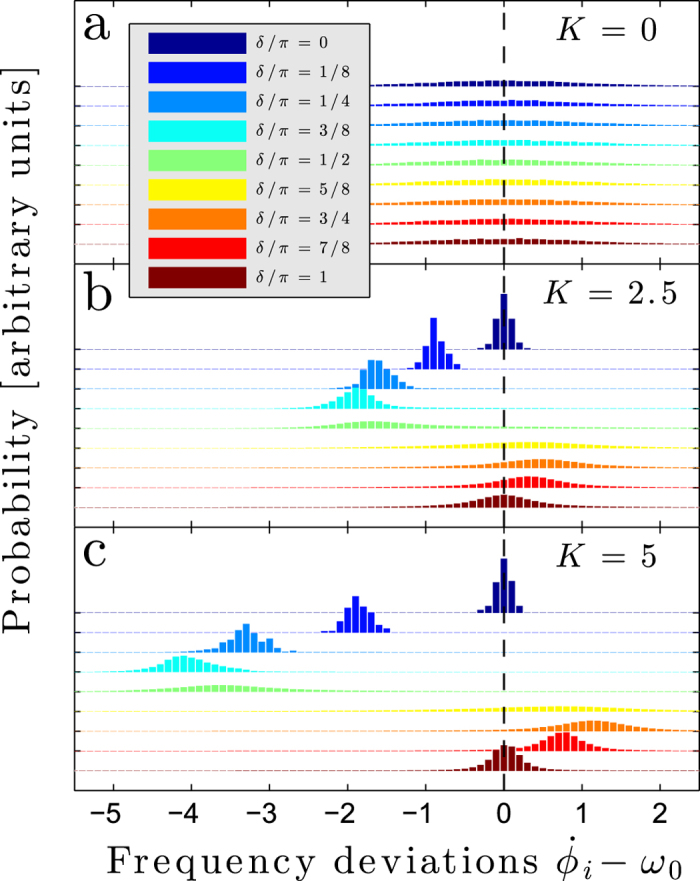
Stationary distributions of frequency deviations 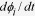 for different values of 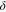 (represented by different colors as indicated in the legend), and for **a** subcritical, **b** weakly and **c** strongly supercritical values of 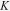, respectively. 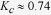 according to Fig. 2. For supercritical 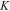, the simultaneous occurrence of rapid frequency suppression and narrowing of the distributions between 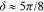 and 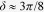 can be observed.

Figure 3a–c shows the stationary distributions of frequency deviations 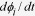 for selected values in the (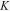,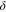)-plane. For 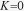, the distributions are practically identical for different values of 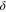, Fig. 3a. For 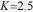, at 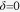, a synchronization peak appears close to frequency zero. With increasing 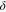, this peak moves towards increasingly negative values, until 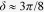. Between 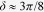 and 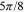, the distribution is rapidly becoming broader and shifts towards positive values. After reaching a maximum at 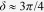, it is finally centered around zero again, Fig. 3b. 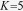, is similar, however larger positive and negative values for 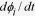 occur, Fig. 3c.

Figure 2d shows the average frequency deviation 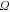 as a function of 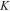 and 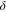. As expected (see SI), we find frequency suppression associated with synchronization in the region of large 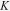 and small 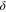, but also for large 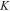 and intermediate 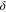. For fixed 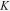, maximal frequency suppression occurs at 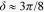. For large 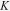 and large 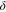 (chaotic region) we find slightly positive 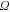.

## Robustness issues

### Homogeneity

The derivation of the MKM is based on three key assumptions, see Eqs. (3)–(5), 35, 41. If Eq. (3) is violated, i.e. the ensemble of uncoupled Wilson-Cowan oscillators is strongly heterogenous, several oscillation periods 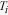 may occur 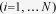. As a consequence, weak interactions become frequency-modulated^27^: Two oscillators interact only if their frequencies 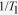 and 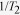 are similar, in the sense that 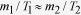, where 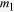 and 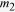 are small numbers.

### Uniqueness of local oscillations

Regarding Eq. (4), discarding the uniqueness of the limit cycles would result in heterogenous coupling strengths 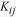, or 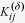.

### Stability of local oscillations and weak coupling

In contrast, both the exponential stability of the limit cycles and the weak coupling assumption, Eq. (5), are strictly necessary for the derivation of the MKM, since they allow for a dimensional reduction from activity- to phase deviation variables (see SI). If the dimensional reduction can not be carried through, the full system Eq. (1) with Eq. (2) has to be studied, whose properties are much harder to access.

### Numerical simulation

We tested the model for robustness with respect to the particular choice of parameters. As suggested by various brain atlases and cortical parcellation schemes, a number of 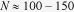 cortical regions seems reasonable^41^,^42^. We tested up to 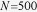 and found no deviations from the presented qualitative picture. For the link density 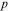, we find that as long as it exceeds the percolation threshold, 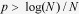, differences in simulations are marginal. Finally, we observe that like in the original Kuramoto model^32^,^33^, for different natural frequency distributions 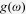 the qualitative behaviour remains practically unchanged as long as 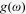 is unimodal and symmetric.

## Discussion

In several variants of single-layer Kuramoto models with a phase shift or a time delay, frequency suppression appears^37^,^39^,^43^,^44^. In addition^39^, mentions chaotic behavior. However, the existence of the phase diagram with the three distinct macroscopic phases can not be inferred from any of those models to the best of our knowledge.

In Ref. 45, several modes of synchronization are reported for a Kuramoto model on two interconnected networks with an inter-network time delay. While this computational model does not exhibit chaotic or unsynchronized phases, it suggests that a more complicated network topology can lead to a deeper structure within the synchronized phase in Kuramoto-type models.

Since the present work emphasizes the derivation of the MKM and the study of its stationary properties, we did not investigate the details of the synchronization transition. In this context we mention that the emergence of synchronization follows different paths in different types of networks^46^. Further, if a correlation between natural frequencies 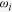 and network properties is assumed, explosive synchronization and hysteretic effects may appear^47^.

Summarizing, we can show mathematically that a set of weakly coupled Wilson-Cowan oscillators on a cortical network with a synaptic time delay between excitatory and inhibitory neural populations is identical to a simple Kuramoto-type phase model on a two-layer multiplex network. Numerical investigations of this model reveal the presence of three distinct macroscopic phases in the space of control parameters 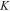 (associated with cerebral blood flow) and 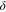 (associated with synaptic GABA concentration). For couplings 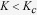, activities of individual cortical regions show independent oscillatory behavior (unsynchronized). Frequencies are distributed symmetrically around an average frequency that we assume to be located in the physiological gamma range. This dynamical state corresponds to “background activity” of the brain. For 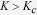, two phases are possible: for small 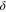, the system becomes synchronized, which corresponds to “epileptic seizure activity” in physiology. For large 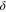, synchronized activity only appears in clusters; the system is chaotic in general. We identify this phase with “resting-state activity” in the brain. An important property of the present model is that the average oscillation frequency is shifted towards lower values when crossing the boundary to the synchronized phase. This could explain the experimental fact^2^,^21^,^22^,^23^ that a decrease of the GABA concentration in the resting-state both triggers the appearance of epileptiform slow waves and diminishes gamma activity in the brain.

## Methods

Equation (6) is integrated with a standard 4th-order Runge-Kutta algorithm with 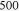 time steps of size 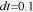. The system size is 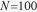, both layers are chosen to be Erdös-Rényi networks with 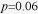. Natural frequencies 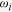 are taken from a standard normal distribution, initial phase deviations 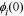 from the interval 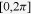. The first 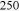 time steps are discarded to exclude transient effects. For the remaining time steps, 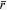, 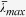, and 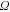 are evaluated. All results are averaged over 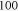 identical, independent runs with different realizations of the initial conditions.

## Additional Information

**How to cite this article**: Sadilek, M. and Thurner, S. Physiologically motivated multiplex Kuramoto model describes phase diagram of cortical activity. *Sci. Rep.* **5,** 10015; doi: 10.1038/srep10015 (2015).

## Supplementary Material

Supporting Information

## References

[b1] Buzsáki G. & Draguhn A. Neuronal oscillations in cortical networks. Science 304, 1926–1929 (2004). 15218136 10.1126/science.1099745

[b2] Eisenstein M. Neurobiology: unrestrained excitement. Nature 511, S4–S6 (2014). 25019125 10.1038/511s4a

[b3] Lewis D.A., Hashimoto T. & Volk D.W. Cortical inhibitory neurons and schizophrenia. Nat. Rev. Neurosci. 6, 312–324 (2005). 15803162 10.1038/nrn1648

[b4] Varela F., Lachaux J.P., Rodriguez E. & Martinerie J. The brainweb: phase synchronization and large-scale integration. Nat. Rev. Neurosci. 2, 229–239 (2001). 11283746 10.1038/35067550

[b5] Stam C. J. Nonlinear dynamical analysis of EEG and MEG: review of an emerging field. J. Clin. Neurophysiol. 116, 2266–2301 (2005). 10.1016/j.clinph.2005.06.011 16115797

[b6] Bullmore E. & Sporns O. Complex brain networks: graph theoretical analysis of structural and functional systems. Nat. Rev. Neurosci. 10, 186–198 (2009). 19190637 10.1038/nrn2575

[b7] Isaacson J. S. & Scanziani M. How inhibition shapes cortical activity. Neuron 72, 231–243 (2011). 22017986 10.1016/j.neuron.2011.09.027 PMC3236361

[b8] Biswal B. B. Resting state fMRI: a personal history. Neuroimage 62, 938–944 (2012). 22326802 10.1016/j.neuroimage.2012.01.090 PMC12911935

[b9] Deco G., Jirsa V. K. & McIntosh A. R. Emerging concepts for the dynamical organization of resting-state activity in the brain. Nat. Rev. Neurosci. 12, 43–56 (2010). 21170073 10.1038/nrn2961

[b10] Honey C. J., Kötter R., Breakspear M. & Sporns O. Network structure of cerebral cortex shapes functional connectivity on multiple time scales. Proc. Natl. Acad. Sci. USA 104, 10240–10245 (2007). 17548818 10.1073/pnas.0701519104 PMC1891224

[b11] Ghosh A., Rho Y., McIntosh A. R., Kötter R. & Jirsa V. K. Noise during rest enables the exploration of the brainÕs dynamic repertoire. PLoS Comput. Biol. 4, e1000196 (2008). 18846206 10.1371/journal.pcbi.1000196 PMC2551736

[b12] Deco G., Jirsa V., McIntosh A. R., Sporns O. & Kötter R. Key role of coupling, delay, and noise in resting brain fluctuations. Proc. Natl. Acad. Sci. USA 106, 10302–10307 (2009). 19497858 10.1073/pnas.0901831106 PMC2690605

[b13] Cabral J., Hugues E., Sporns O. & Deco G. Role of local network oscillations in resting-state functional connectivity. Neuroimage 57, 130–139 (2011). 21511044 10.1016/j.neuroimage.2011.04.010

[b14] Singer W. & Gray C. M. Visual feature integration and the temporal correlation hypothesis. Annu. Rev. Neurosci. 18, 555–586 (1995). 7605074 10.1146/annurev.ne.18.030195.003011

[b15] Buzsáki G. & Wang X. J. Mechanisms of gamma oscillations. Annu. Rev. Neurosci. 35, 203–225 (2012). 22443509 10.1146/annurev-neuro-062111-150444 PMC4049541

[b16] Whittington M. A., Traub R. D. & Jefferys J. G. R. Synchronized oscillations in interneuron networks driven by metabotropic glutamate receptor activation. Nature 373, 612–615 (1995). 7854418 10.1038/373612a0

[b17] Wang X. J. & Buzsáki G. Gamma oscillation by synaptic inhibition in a hippocampal interneuronal network model. J. Neurosci. 16, 6402–6413 (1996). 8815919 10.1523/JNEUROSCI.16-20-06402.1996 PMC6578902

[b18] Brunel N. & Wang X.J. What determines the frequency of fast network oscillations with irregular neural discharges? I. Synaptic dynamics and excitation-inhibition balance. J. Neurophysiol. 90, 415–430 (2003). 12611969 10.1152/jn.01095.2002

[b19] Geisler C., Brunel N. & Wang X. J. Contributions of intrinsic membrane dynamics to fast network oscillations with irregular neuronal discharges. J. Neurophysiol. 94, 4344–4361 (2005). 16093332 10.1152/jn.00510.2004

[b20] Wilson H. R. & Cowan J. D. Excitatory and inhibitory interactions in localized populations of model neurons. Biophys. J. 12, 1–24 (1972). 4332108 10.1016/S0006-3495(72)86068-5 PMC1484078

[b21] Mann E. O. & Mody I. Control of hippocampal gamma oscillation frequency by tonic inhibition and excitation of interneurons. Nat. Neurosci. 13, 205–212 (2010). 20023655 10.1038/nn.2464 PMC2843436

[b22] Medvedev A. V. Epileptiform spikes desynchronize and diminish fast (gamma) activity of the brain: an “anti-binding” mechanism? Brain. Res. Bull. 58, 115–128 (2002). 12121821 10.1016/s0361-9230(02)00768-2

[b23] Muthukumaraswamy S. D., Edden R. A. E., Jones D. K., Swettenham J. B. & Singh K. D. Resting GABA concentration predicts peak gamma frequency and fMRI amplitude in response to visual stimulation in humans Proc. Natl. Acad. Sci. USA 106, 8356–8361 (2009). 19416820 10.1073/pnas.0900728106 PMC2688873

[b24] Boccaletti S. *et al.* The structure and dynamics of multilayer networks. Phys. Rep . 544, 1–122 (2014). 10.1016/j.physrep.2014.07.001 PMC7332224 32834429

[b25] Wang Z., Szolnoki A. & Perc M. Evolution of public cooperation on interdependent networks: the impact of biased utility functions. Europhys. Lett. 97, 48001 (2012).

[b26] Borisyuk R. M. & Kirillov A. B. Bifurcation analysis of a neural network model. Biol. Cybern. 66, 319–325 (1992). 1550881 10.1007/BF00203668

[b27] Hoppensteadt F.C. & Izhikevich E.M. Weakly Connected Neural Networks (Springer: New York, 1997).

[b28] Fairen A., DeFelipe J. & Regidor J. Nonpyramidal neurons: general account. Cereb. Cortex 1, 201–253 (1984).

[b29] DeFelipe J. & Fariñas I. The pyramidal neuron of the cerebral cortex: morphological and chemical characteristics of the synaptic inputs. Prog. Neurobiol. 39, 563–607 (1992). 1410442 10.1016/0301-0082(92)90015-7

[b30] Tsurugizawa T., Ciobanu L. & Le Bihan D. Water diffusion in brain cortex closely tracks underlying neuronal activity. Proc. Natl. Acad. Sci. USA 110, 11636–11641 (2013). 23801756 10.1073/pnas.1303178110 PMC3710802

[b31] Winfree A. Biological rhythms and the behavior of populations of coupled oscillators. J. Theor. Biol. 16, 15–42 (1967). 6035757 10.1016/0022-5193(67)90051-3

[b32] Kuramoto Y. [Self-entrainment of a population of coupled non-linear oscillators] International Symposium On Mathematical Problems In Theoretical Physics [ Araki H. (ed.)] (Springer: Berlin Heidelberg, 1975).

[b33] Kuramoto Y. Cooperative dynamics of oscillator community. Prog. Theor. Phys. Supp. 79, 223–240 (1984).

[b34] Arenas A., Díaz-Guilera A., Kurths J., Moreno Y. & Zhou C. Synchronization in complex networks. Phys. Rep . 469, 93–153 (2008).

[b35] Kalloniatis A. C. From incoherence to synchronicity in the network Kuramoto model. Phys. Rev. E 82, 066202 (2010). 10.1103/PhysRevE.82.066202 21230718

[b36] Miritello G., Pluchino A. & Rapisarda A. Central limit behavior in the Kuramoto model at the “edge of chaos”. Physica A 388, 4818–4826 (2009).

[b37] Niebur E., Schuster H. G. & Kammen D. M. Collective frequencies and metastability in networks of limit-cycle oscillators with time delay. Phys. Rev. Lett. 67, 2753 (1991). 10044546 10.1103/PhysRevLett.67.2753

[b38] Yeung M. K. S. & Strogatz S. H. Time delay in the Kuramoto model of coupled oscillators. Phys. Rev. Lett. 82, 648 (1999).

[b39] Nicosia V., Valencia M., Chavez M., Díaz-Guilera A. & Latora V. Remote synchronization reveals network symmetries and functional modules. Phys. Rev. Lett. 110, 174102 (2013). 23679731 10.1103/PhysRevLett.110.174102

[b40] Acebrón J. A., Bonilla L. L., Vicente C. J. P., Ritort F. & Spigler R. The Kuramoto model: a simple paradigm for synchronization phenomena. Rev. Mod. Phys. 77, 137–185 (2005).

[b41] Zilles K. & Amunts K. Centenary of Brodmanns map conception and fate. Nat. Rev. Neurosci. 11, 139–145 (2010). 20046193 10.1038/nrn2776

[b42] Van Essen D. C., Glasser M. F., Dierker D. L., Harwell J. & Coalson T. Parcellations and hemispheric asymmetries of human cerebral cortex analyzed on surface-based atlases. Cereb. Cortex 22, 2241–2262 (2011). 22047963 10.1093/cercor/bhr291 PMC3432236

[b43] Louzada V. H. P., Araújo N. A. M., Andrade J. S. Jr. & Herrmann H. J. How to suppress undesired synchronization. Sci. Rep . 2, 658 (2012). 22993685 10.1038/srep00658 PMC3443817

[b44] Choi M. Y., Kim H. J., Kim D. & Hong H. Synchronization in a system of globally coupled oscillators with time delay. Phys. Rev. E 61, 371 (2000). 10.1103/physreve.61.371 11046275

[b45] Louzada V. H. P., Araújo N. A. M., Andrade J.S. Jr. & Herrmann H.J. Breathing synchronization in interconnected networks. Sci. Rep. 3, 3289 (2013). 24256765 10.1038/srep03289 PMC3836035

[b46] Gómez-Gardeñes J., Moreno Y. & Arenas A. Paths to synchronization on complex networks. Phys. Rev. Lett. 98, 034101 (2007). 17358685 10.1103/PhysRevLett.98.034101

[b47] Sendiña-Nadal I. *et al.* Assortative mixing enhances the irreversible nature of explosive synchronization in growing scale-free networks. arXiv:1408.2194 (2014).

[b48] Strogatz S. H. Nonlinear Dynamics And Chaos: With Applications To Physics, Biology, Chemistry, And Engineering (Perseus Books Group: New York, 1994).

