# Supplementary material for: Physiologically motivated multiplex Kuramoto model describes phase diagram of cortical activity
Source: Sci Rep. 2015 May 21;5:10015. doi: 10.1038/srep10015 (PMC4650820; doi:10.1038/srep10015)
Supplement: Supporting Information [file srep10015-s1.pdf]

Supplementary Information: Physiologically motivated multiplex Kuramoto model describes phase diagram of cortical activity

Maximilian Sadilek[1] and Stefan Thurner[1,2,3]

[1]Section for Science of Complex Systems, Medical University of Vienna, Spitalgasse 23, A-1090, Vienna, Austria
[2]Santa Fe Institute, 1399 Hyde Park Road, New Mexico 87501, USA
[3]IIASA, Schlossplatz 1, A-2361 Laxenburg; Austria

DERIVATION OF THE MULTIPLEX KURAMOTO MODEL

In this section, we derive the MKM, Eq. (6) in the main text, from nearly identical, weakly coupled Wilson-Cowan oscillators, see Eqs. (1) – (5). The main idea is the following: Starting from uncoupled oscillators having exponentially stable orbits, the introduction of weak coupling between them only affects their phases (and not their frequencies or amplitudes). This allows for a transformation from activity- to phase deviation variables. The special structure of the Wilson-Cowan equations then accounts for the multilayer structure of the MKM.

For compact notation, we define $\boldsymbol{X}_i \equiv (x_i, y_i)^\top$ and $\boldsymbol{X} \equiv (\boldsymbol{X}_1, \dots \boldsymbol{X}_N)$, where $^\top$ denotes transposition. Assumptions expressed in Eqs. (3) and (5), allow us to write Eq. (1) with the interactions introduced by Eq. (2) in the form

$$\frac{d\boldsymbol{X}_i(t)}{dt} = \boldsymbol{F}\left[\boldsymbol{X}_i(t)\right] + \varepsilon\, \delta\boldsymbol{F}_i\left[\boldsymbol{X}_i(t)\right] + \varepsilon\, \boldsymbol{G}_i\left[\boldsymbol{X}(t), \boldsymbol{X}(t-\tau)\right] \quad , \tag{S1}$$

up to terms of order $\mathcal{O}(\varepsilon^2)$. Here $\boldsymbol{F}$ constitutes the uncoupled part of the dynamics, and $\delta\boldsymbol{F}_i$ accounts for small perturbations in the parameters $a, b, c, d, \rho^{(x)}$, and $\rho^{(y)}$. $\boldsymbol{G}_i$ encodes the contributions from weak coupling. Terms of order $\mathcal{O}(\varepsilon^2)$ will be omitted in all subsequent expressions.

Assumption from Eq. (4) states that solutions $\boldsymbol{X}_i^*(t)$ of the uncoupled system

$$\frac{d\boldsymbol{X}_i(t)}{dt} = \boldsymbol{F}\left[\boldsymbol{X}_i(t)\right]$$

approach a unique, exponentially stable limit cycle $\gamma \subset \mathbb{R}^2$. This means that after a transient phase we can write $\boldsymbol{X}_i^*(t) = \boldsymbol{\Gamma}(t + \varphi_i)$. We define the phase of such a solution by the mapping $\theta\colon \boldsymbol{X}_i^*(t) \mapsto 2\pi/T(t+\varphi_i) \in \mathcal{S}^1$, uniquely assigning a point on the unit cycle to each point of the solution, where T is the period of $\boldsymbol{\Gamma}$.

Since solutions $\boldsymbol{X}_i^*(t)$ of the uncoupled system are exponentially orbitally stable, we can write solutions of the weakly coupled system Eq. (S1) as

$$\boldsymbol{X}_i(t) = \boldsymbol{\Gamma}\left[t + \frac{T}{2\pi}\phi_i(\varepsilon t)\right] + \varepsilon \boldsymbol{P}_i\left[t + \frac{T}{2\pi}\phi_i(\varepsilon t)\right] \quad , \tag{S2}$$

where $\phi_i(t) \in \mathcal{S}^1$ is the deviation from the phase $\theta_i(t) \equiv \theta(\boldsymbol{X}_i^*(t)) = 2\pi/T(t+\varphi_i)$, see Fig. 1(c) in the main text. $\varepsilon\boldsymbol{P}_i$ accounts for the effects of the ε-perturbation of the invariant manifold $\gamma^N \subset \mathbb{R}^{2N}$, where γ^N denotes the N-th Cartesian power of γ, [S1]. Initial values of ϕ_i are given by

$$\phi_i(0) = \frac{2\pi}{T}\varphi_i \quad .$$

Differentiating Eq. (S2) with respect to t and using Eq. (S1), we arrive at the linear inhomogeneous equation

$$\frac{d\boldsymbol{v}_i(t,\phi_i)}{dt} = \boldsymbol{M}(t,\phi_i)\, \boldsymbol{v}_i(t,\phi_i) + \boldsymbol{m}_i(t,\boldsymbol{\phi}) \quad , \tag{S3}$$

where $\boldsymbol{\phi} \equiv (\phi_1(\varepsilon t), \dots \phi_N(\varepsilon t))$ and

$$\boldsymbol{v}_i(t,\phi_i) \equiv \boldsymbol{P}_i\left(t + \frac{T}{2\pi}\phi_i\right) \quad ,$$

$$\boldsymbol{M}(t,\phi_i) \equiv D\boldsymbol{F}\left(t + \frac{T}{2\pi}\phi_i\right) \quad ,$$

$$\boldsymbol{m}_i(t,\boldsymbol{\phi}) \equiv \boldsymbol{G}_i\left(t + \frac{T}{2\pi}\boldsymbol{\phi}, t - \tau + \frac{T}{2\pi}\boldsymbol{\phi}\right) + \delta\boldsymbol{F}_i\left(t + \frac{T}{2\pi}\phi_i\right) - \boldsymbol{F}\left(t + \frac{T}{2\pi}\phi_i\right)\frac{d\phi_i}{d(\varepsilon t)} \quad .$$

Here DF denotes the Jacobian matrix of $\boldsymbol{F}$, and we have used the abbreviations $\boldsymbol{F}(t) \equiv \boldsymbol{F}(\boldsymbol{\Gamma}(t))$, $\delta\boldsymbol{F}_i(t) \equiv \delta\boldsymbol{F}_i(\boldsymbol{\Gamma}(t))$, and $\boldsymbol{G}_i(t, t-\tau) \equiv \boldsymbol{G}_i(\boldsymbol{\Gamma}(t), \boldsymbol{\Gamma}(t-\tau))$ for clarity of notation. Now, since both solutions $\boldsymbol{v}_i(t, \phi_i)$ of (S3) and $\boldsymbol{w}(t, \phi_i)$ of the adjoint *homogeneous* system

$$\frac{d\boldsymbol{w}(t,\phi_i)}{dt} = -\boldsymbol{M}(t,\phi_i)^\top \boldsymbol{w}(t,\phi_i) \tag{S4}$$

are periodic, nontrivial and unique by construction, the orthogonality condition

$$\frac{1}{T}\int_0^T \boldsymbol{w}(t,\phi_i)^\top \boldsymbol{m}_i(t,\boldsymbol{\phi})\, dt = 0 \quad , \tag{S5}$$

follows (Fredholm alternative). If we normalize the solution $\boldsymbol{w}(t,\phi_i)$ of Eq. (S4) by the condition

$$\frac{1}{T}\int_0^T \boldsymbol{w}(t,\phi_i)^\top \frac{T}{2\pi}\boldsymbol{F}\left(t + \frac{T}{2\pi}\phi_i\right) dt = 1 \quad , \tag{S6}$$

insert the definition of $\boldsymbol{m}_i(t,\boldsymbol{\phi})$ into Eq. (S5), and use Eq. (S6) we obtain

$$\frac{d\phi_i}{d(\varepsilon t)} = \frac{1}{T}\int_0^T \boldsymbol{w}(t,\phi_i)^\top \left[\delta\boldsymbol{F}_i\left(t + \frac{T}{2\pi}\phi_i\right) + \boldsymbol{G}_i\left(t + \frac{T}{2\pi}\boldsymbol{\phi}, t - \tau + \frac{T}{2\pi}\boldsymbol{\phi}\right)\right] dt \quad .$$

Due to Eq. (S4) and the special form of $\boldsymbol{M}(t,\phi_i)$ we have $\boldsymbol{w}(t,\phi_i) = \boldsymbol{w}\left(t + \frac{T}{2\pi}\phi_i, 0\right)$. If we define $\boldsymbol{Q}(t) \equiv \boldsymbol{w}(t,0)$ and make the substitutions $t \to t + \frac{T}{2\pi}\phi_i$ and $\varepsilon t \to t$, we obtain

$$\frac{d\phi_i}{dt} = \frac{1}{T}\int_0^T \boldsymbol{Q}(t)^\top \left\{\delta\boldsymbol{F}_i\left[\boldsymbol{\Gamma}\left(t\right)\right] + \boldsymbol{G}_i\left[\boldsymbol{\Gamma}\left(t + \frac{T}{2\pi}(\boldsymbol{\phi} - \phi_i)\right), \boldsymbol{\Gamma}\left(t - \tau + \frac{T}{2\pi}(\boldsymbol{\phi} - \phi_i)\right)\right]\right\} dt \quad , \tag{S7}$$

where we expanded the shortened notation for $\boldsymbol{F}$, $\delta\boldsymbol{F}_i$ and $\boldsymbol{G}_i$ again. Making use of the structure of $\boldsymbol{G}_i$, see Eqs. (1), (2), (S1), we can rewrite Eq. (S7) as

$$\frac{d\phi_i}{dt} = \omega_i + \sum_{j\neq i}^{N}\Big[A_{ij}H_{EE}(\phi_j - \phi_i) + B_{ij}H_{EI}(\phi_j - \phi_i - \delta) + C_{ij}H_{IE}(\phi_j - \phi_i - \delta)\Big] \quad , \tag{S8}$$

where δ is a phase shift parameter related to the time delay τ through $\delta = (2\pi/T)\tau$, and

$$\begin{aligned}
\omega_i &\equiv \frac{1}{T}\int_0^T \boldsymbol{Q}(t)^\top \delta\boldsymbol{F}_i\left[\boldsymbol{\Gamma}(t)\right] dt \quad , \\
H_{EE}(\chi) &\equiv \frac{1}{T}\int_0^T Q_1(t) S'\left(ax(t) - by(t) + \rho^{(x)}\right) x\left(t + \frac{T}{2\pi}\chi\right) dt \quad , \\
H_{EI}(\chi) &\equiv -\frac{1}{T}\int_0^T Q_1(t) S'\left(ax(t) - by(t) + \rho^{(x)}\right) y\left(t + \frac{T}{2\pi}\chi\right) dt \quad , \\
H_{IE}(\chi) &\equiv \frac{1}{T}\int_0^T Q_2(t) S'\left(cx(t) - dy(t) + \rho^{(y)}\right) x\left(t + \frac{T}{2\pi}\chi\right) dt \quad .
\end{aligned}$$

Here $S'(x) \equiv dS/du|_{u=x}$, Q_1 and Q_2 are the two components of $\boldsymbol{Q}$, and $x\left(t + \frac{T}{2\pi}\chi\right)$ and $y\left(t + \frac{T}{2\pi}\chi\right)$ are the two components of $\boldsymbol{\Gamma}\left(t + \frac{T}{2\pi}\chi\right)$, respectively. Following the argument of Kuramoto [S2, S3], we keep only the sinusoidal terms of a Fourier series expansion of the right hand side of Eq. (S8), and obtain

$$\frac{d\phi_i}{dt} = \omega_i + \sum_{j\neq i} K_A A_{ij}\sin(\phi_j - \phi_i) + \sum_{j\neq i} K_A^{(\delta)} A_{ij}^{(\delta)}\sin(\phi_j - \phi_i - \delta) \quad , \tag{S9}$$

where $A_{ij}^{(\delta)}$ is a linear combination of B_{ij} and C_{ij}, and K_A and $K_A^{(\delta)}$ are constants. Without loss of generality we assume that K_A and $K_A^{(\delta)}$ have equal signs. This is valid because the replacement

$$(K_A^{(\delta)}, \delta) \to (-K_A^{(\delta)}, \delta - \pi)$$

leaves Eq. (S9) invariant. We further assume that

$$K_A \langle k \rangle = K_A^{(\delta)} \left\langle k^{(\delta)} \right\rangle \equiv K \quad , \tag{S10}$$

where $\langle k \rangle \equiv 1/N \sum_{i,j}^{N} A_{ij}$ and $\left\langle k^{(\delta)} \right\rangle \equiv 1/N \sum_{i,j}^{N} A_{ij}^{(\delta)}$ are the average degrees of the excitatory-excitatory and excitatory-inhibitory networks, respectively. Eq. (S10), together with assumption (*ii*) in the main text, constrains the possible range of parameters $\left(a, b, c, d, \rho^{(x)}, \rho^{(y)}, A_{ij}, B_{ij}, C_{ij}\right)$. To justify Eq. (S10), we note that $d\phi_i/dt \in \left[\omega_i - \omega_i^{int}, \omega_i + \omega_i^{int}\right]$, where

$$\omega_i^{int} \equiv \sum_{j \neq i} \left(|K_A| \, A_{ij} + \left| K_A^{(\delta)} \right| A_{ij}^{(\delta)} \right) \quad .$$

Hence, we can estimate the maximum rate at which system Eq. (S9) relaxes to a stationary state, characterized by $d\phi_i/dt = 0$, by

$$\Omega^{\max} \equiv \frac{1}{N} \sum_i \left(\frac{d\phi_i}{dt} \right)^{\max} = \frac{1}{N} \sum_i \left(|\omega_i| + \omega_i^{int} \right) = \frac{1}{N} \sum_i |\omega_i| + |K_A| \langle k \rangle + \left| K_A^{(\delta)} \right| \left\langle k^{(\delta)} \right\rangle \quad .$$

We see that Eq. (S10) implies that both types of interaction contribute equally to the relaxation rate. This is consistent with the property of the Wilson-Cowan model that the time scale associated with the physiological response of a population of neurons to either excitatory or inhibitory activity of adjacent populations does not depend on the input type [S4]. With this notation we obtain the two-layer MKM, i.e. Eq. (6) in the main text,

$$\frac{d\phi_i}{dt} = \omega_i + \frac{K}{\langle k \rangle} \sum_{j=1}^{N} A_{ij} \sin(\phi_j - \phi_i) + \frac{K}{\left\langle k^{(\delta)} \right\rangle} \sum_{j=1}^{N} A_{ij}^{(\delta)} \sin(\phi_j - \phi_i - \delta) \quad .$$

THE CASE $\delta = 0$

In this section, we investigate some of the properties of the single-layer Kuramoto model,

$$d\phi_i/dt = \omega_i + K/\langle k \rangle \sum_{j=1}^{N} \alpha_{ij} \sin(\phi_j - \phi_i) \quad , \tag{S11}$$

that is obtained by setting $\delta = 0$ in Eq. (6), and introducing the abbreviation $\alpha_{ij} \equiv A_{ij} + A_{ij}^{(\delta)} \langle k \rangle / \left\langle k^{(\delta)} \right\rangle$.

Synchronization. For sufficiently large N and sufficiently dense networks A_{ij} and $A_{ij}^{(\delta)}$, we expect the following behavior [S5, S6]: For small values of K, the system attains a stationary global state that shows no synchronization, $\overline{r} \sim 0$, up to finite-size fluctuations of order $\mathcal{O}(1/\sqrt{N})$. Above a critical value,

$$K_c \equiv K_c^0 \langle k \rangle \frac{\langle \kappa \rangle}{\langle \kappa^2 \rangle} \quad , \tag{S12}$$

synchronized clusters appear, $\overline{r} > 0$, where $K_c^0 \equiv 2/(\pi g(0))$, is the critical value for a fully connected network, and $\langle \kappa \rangle$ and $\langle \kappa^2 \rangle$ are the first two moments of the distribution of degrees $\kappa_i \equiv \sum_{j=1}^{N} \alpha_{ij}$, respectively. If both A_{ij} and $A_{ij}^{(\delta)}$ are Erdős-Rényi networks with probability p, κ_i follows a binomial distribution with probability $2p$. Hence we get $K_c = K_c^0/2\,(1 - (1-2p)/2pN) + \mathcal{O}\left(N^{-2}\right)$, meaning that the Kuramoto transition occurs at slightly lower values than for fully connected networks, as long as $\log(N)/N < p < 0.5$. For $K >> K_c$, global synchronization is attained, $\overline{r} \sim 1$, after a transient time $\propto \langle k \rangle / (K\lambda_2)$, where λ_2 is the second-smallest eigenvalue of the Laplacian, $L_{ij} \equiv \kappa_i \delta_{ij} - \alpha_{ij}$.

Chaotic dynamics. The largest Lyapunov exponent takes the value $\overline{\lambda}_{max} = 0$ in the subcritical regime, and becomes positive around $K = K_c$, reaching peak values of $\overline{\lambda}_{max} \approx 0.05$. For $K > K_c$, $\overline{\lambda}_{max}$ decreases to marginally negative values $\overline{\lambda}_{max} \approx -0.001$, [S7].

Average frequency deviation. For large values of K, solutions of Eq. (S11) are globally synchronized, and $\Omega = 1/N \sum_{i=1}^{N} \omega_i \approx \omega_0$. Related models [S8–S10] suggest that small phase shifts $\delta > 0$ do not destroy synchronization, so we expect $\Omega \approx \omega_0 - K/(\left\langle k^{(\delta)} \right\rangle N) \sum_{i,j} A_{ij}^{(\delta)} \cos(\phi_j - \phi_i) \sin(\delta) \approx \omega_0 - K\delta$, i.e. frequency suppression, for large values of K and small values of δ. Here we used properties of the sine function and of the synchronized solution.

CALCULATION OF THE INSTANTANEOUS LARGEST LYAPUNOV EXPONENT

In this section, we describe the algorithm that we use to calculate estimates for the instantaneous largest Lyapunov exponent $\lambda_{max}(t)$ together with the numerical integration of Eq. (6). Since solutions of Eq. (6) are vectors of phases $\boldsymbol{\phi} = (\phi_1, \ldots \phi_N)$, an appropriate distance function $d(\boldsymbol{\phi}, \boldsymbol{\psi})$ on the N-torus, measuring the separation between reference orbits and perturbed orbits at each time step, is needed. We choose the 1-norm distance

$$d(\boldsymbol{\phi}, \boldsymbol{\psi}) = \sum_{i=1}^{N} |\phi_i - \psi_i| \quad , \tag{S13}$$

since it maximizes all p-norm distances, thus providing an upper bound on the separation distance. Note that making use of the embedding into Euclidean space $\boldsymbol{\phi} \rightarrow (\sin(\boldsymbol{\phi}), \cos(\boldsymbol{\phi}))$ together with applying the 2-norm runs into obvious problems in the renormalization step of the algorithm (see below).

An initial separation between the reference trajectory and the perturbed trajectory of $d_0 = 10^{-4}$ is chosen. Both trajectories are integrated for one time step $dt = 0.1$ according to Eq. (6), and the quantity $\lambda_1 = \ln(d_1/d_0)/dt$ is calculated. d_1 is the separation between the trajectories after one step. For small initial separations d_0 this quantity is a good approximation for the instantaneous Lyapunov exponent after one time step [S11]. Now the perturbed trajectory is renormalized in the direction of the difference vector to the reference trajectory to have a separation of d_0 again. After another integration step the quantity $\lambda_2 = \ln(d_2/d_0) + \ln(d_1/d_0)/(2dt)$ is calculated and the perturbed trajectory is renormalized as before. Iteration yields estimates for the instantaneous largest Lyapunov exponents

$$\lambda_{max}(t_n) = \lambda_n = \frac{1}{t_n - t_0} \sum_{i=1}^{n} \ln\left(\frac{d_i}{d_0}\right) \quad . \tag{S14}$$

The algorithm was tested in a discrete version with the logistic map; $d_0 = 10^{-4}$ turned out to be sufficiently small to provide satisfactory results.

[S1] Hoppensteadt, F.C. & Izhikevich, E.M. *Weakly Connected Neural Networks* (Springer, New York, 1997).

[S2] Kuramoto, Y. [Self-entrainment of a population of coupled non-linear oscillators] *International Symposium On Mathematical Problems In Theoretical Physics* [Araki, H. (ed.)] (Springer, Berlin Heidelberg, 1975).

[S3] Kuramoto, Y. Cooperative dynamics of oscillator community. *Prog. Theor. Phys. Supp.* **79,** 223-240 (1984).

[S4] Wilson, H.R. & Cowan, J.D. Excitatory and inhibitory interactions in localized populations of model neurons. *Biophys. J.* **12,** 1-24 (1972).

[S5] Arenas, A., Díaz-Guilera, A., Kurths, J., Moreno, Y. & Zhou, C. Synchronization in complex networks. *Phys. Rep.* **469,** 93-153 (2008).

[S6] Kalloniatis, A.C. From incoherence to synchronicity in the network Kuramoto model. *Phys. Rev. E* **82,** 066202 (2010).

[S7] Miritello, G., Pluchino, A. & Rapisarda, A. Central limit behavior in the Kuramoto model at the "edge of chaos". *Physica A* **388,** 4818-4826 (2009).

[S8] Niebur, E., Schuster, H.G. & Kammen, D.M. Collective frequencies and metastability in networks of limit-cycle oscillators with time delay. *Phys. Rev. Lett.* **67,** 2753 (1991).

[S9] Yeung, M.K.S. & Strogatz, S.H. Time delay in the Kuramoto model of coupled oscillators. *Phys. Rev. Lett.* **82,** 648 (1999).

[S10] Nicosia, V., Valencia, M., Chavez, M., Díaz-Guilera, A. & Latora, V. Remote synchronization reveals network symmetries and functional modules. *Phys. Rev. Lett.* **110,** 174102 (2013).

[S11] Strogatz, S.H. *Nonlinear Dynamics And Chaos: With Applications To Physics, Biology, Chemistry, And Engineering* (Perseus Books Group, New York, 1994).
